# Do north-eastern German pharmacies recommend a necessary medical consultation for acute diarrhoea? Magnitude and determinants using a simulated patient approach

**DOI:** 10.12688/f1000research.21045.2

**Published:** 2020-05-07

**Authors:** Bernhard Langer, Christian Kunow

**Affiliations:** 1 Department of Health, Nursing, Management, University of Applied Sciences Neubrandenburg, Neubrandenburg, Germany

**Keywords:** Non-prescription drugs, Community pharmacies, Consultation, Patient simulation, Diarrhoea, Germany

## Abstract

**Background:** In Germany, non-pharmacists (pharmacy technicians and pharmaceutical technical assistants) are permitted to advise on and sell medications in addition to pharmacists. The aim of this study was to determine if pharmacists and non-pharmacists referred patients to a medical consultation for a scenario in which consulting a doctor was mandatory (‘appropriate outcome’) and what the quality of questioning and – if a medication was dispensed – the quality of information provided were in this context. The study also aimed to determine which factors predicted a necessary referral to a doctor.

**Methods:** A cross-sectional, covert simulated patient study was conducted in a random sample of community pharmacies stratified by location in the German state of Mecklenburg-Vorpommern. Each pharmacy was visited once by one of four trained investigators. They simulated a symptom-based request involving a grandmother with acute diarrhoea. A multivariate binary logistic regression analysis using potential variables from bivariate analysis was carried out to determine the predictors for a referral to a doctor.

**Results:** All 199 planned visits were conducted. A necessary referral to a doctor was recommended in 59.8% (n=119) of all visits. The most commonly asked question was ‘for whom is the medication?’ (75.4%, n=150), while ‘clarification by a doctor’ was asked the least (17.6%, n=35). In 87.9% (n=175) of all visits a medication was dispensed. Multivariate analysis revealed that, unlike pharmacists, non-pharmacists have a 2.446 times higher likelihood of recommending a referral to a doctor (p = 0.044; 95% CI = 1.025–5.835).

**Conclusions:** In almost half of the visits a necessary referral to a doctor was not recommended. Furthermore, the quality of questioning and the quality of information were below expectations. Moreover, involvement of non‑pharmacists was surprisingly identified as a relevant factor influencing the appropriate outcome.

## Introduction

Acute diarrhoea is one of the most common diseases worldwide ^1^, including in Germany ^2^. Worldwide about 1 billion people develop acute diarrhoea each year ^2^, causing about 2 billion cases of acute diarrhoea ^3^, and about 30% of the German population suffer from acute diarrhoea episodes each year ^2^. Because those affected report becoming ill on average 1.7 times per year ^2^, this means that about 42 million cases of acute diarrhoea can therefore be expected in Germany.

According to the guidelines of the German Society of General Medicine and Family Medicine, acute diarrhoea is an imbalance between secretion and absorption in the bowels, whereby the symptoms last fewer than 14 days and are associated with an increased frequency (≥ three loose stools in a day) or an increased water content (≥ 75%) or an increased stool weight (≥ 250 g) ^4^.

Acute diarrhoea may be a result of infections with viruses (for example, noroviruses or rotaviruses), bacteria (for example, non-typhoidal *Salmonella* or diarrheagenic *Escherichia coli*) or parasites (for example, *Cryptosporidium parvum*) ^5^. More than 90% of cases result from infections, with viral infections being the most common cause ^6^. Bacterial infections, on the other hand, are more often associated with travel, food poisoning and comorbidities. Medications (e.g. antibiotics, cytostatics, diuretics), gastrointestinal disorders (e.g. ulcerative colitis, Crohn’s disease), endocrine disorders (e.g. hyperthyroidism, adrenocortical insufficiency) or other medical conditions (e.g. amyloidosis) can also trigger acute diarrhoea ^5^.

In Germany there is no medical consultation in about two-thirds of cases of acute diarrhoea ^2^. Therefore, advice should be provided by community pharmacies (CP), not least because of the wide range of possible causes, when the diarrhoea is self-medicated with over-the-counter (OTC) medications that are only available from CPs in Germany. As part of a high quality consultation, pharmacy staff should also identify when it is necessary to refer a patient to a medical consultation based on symptoms ^7, 8^. In Germany, non-pharmacists (pharmacy technicians and pharmaceutical technical assistants) are permitted to advise on and sell medications in addition to pharmacists.

Although studies available from Germany regarding the quality of advice provided in CPs reveal significant deficits, analogous to the international literature ^9– 12^, indications other than acute diarrhoea in adults have been investigated in scientific ^13, 14^ and non-scientific studies ^15– 19^. Because of this lack of studies specifically for acute diarrhoea, the quality of advice provided for acute diarrhoea in adults was analysed for Germany for the first time in 2014 and also identified clear deficiencies ^20^. Despite performance feedback used in the process to improve the quality of advice, a follow-up study in 2017 identical to that conducted in 2014 again showed poor quality of advice ^21, 22^. All the above-mentioned studies have in common that they are based on the simulated patient method (SPM) to determine the quality of advice provided. The SPM is a covert participatory observation by a person who, in an ideal situation, is indistinguishable from a real customer (simulated patient, SP) and visits a CP to simulate a real-life consulting situation based on a previously defined scenario. The data are then collected on the basis of previously defined criteria using an assessment form and the CP is provided with performance feedback, if applicable ^23^.

Despite the two studies from 2014 and 2017 mentioned above, there remains a lack of information about the quality of advice for the indication acute diarrhoea in Germany. The reason is that the four scenarios investigated in these two studies were designed as ‘moderate’, in that referral to a medical consultation by the pharmacy staff was not mandatory in any of the scenarios. Both studies also investigated only the quality of advice provided in 21 CPs in a medium-sized northern German city (a total of 84 visits for each study).

The aim of this current study was, therefore, to determine, on the basis of a considerably larger number of CPs, whether pharmacy staff referred patients to a medical consultation for a scenario in which consulting a doctor was mandatory (‘appropriate outcome’) and what the quality of questioning and – if a medication was dispensed – the quality of information provided were in this context. Factors influencing the ‘appropriate outcome’ were also determined analogous to other national and international studies ^14, 21, 24– 27^.

## Methods

### Design

A cross-sectional study design was chosen in accordance with the ‘STROBE Statement – Checklist of items that should be included in reports of cross-sectional studies’ ^28^ and, to determine the quality of advice provided in the CPs investigated, based on the highly recommended ^29^ SP method that is often used internationally ^23, 30– 33^ as a form of participatory observation ^34^. It must once more be explicitly emphasized that this study shares no similarities with the previous studies conducted in 2014 and 2017 apart from the indication investigated (acute diarrhoea).

### Setting and participation

Because of time constraints associated with the SPs, the visits took place over winter between 1 November and 15 December 2018 in the German state of Mecklenburg-Vorpommern (31 December 2018: approx. 1.60 million residents; 23,216 km ^2^ area; low population density of 68.9 residents/km ^2^) ^35^. The Pharmacy Association of Mecklenburg-Vorpommern declined to provide a list of all registered CPs in the state when requested by phone. Consequently, CPs were identified using the pharmacy finder available on the website Apotheken-Umschau.de ^36^. All CPs that had a postcode in the state of Mecklenburg-Vorpommern on the reference date of 1 October 2018 using the postcode search of the pharmacy finder were included in the study. These hits were validated with a corresponding Google search. As a result, a basic population of *N*=396 CPs was determined. A comparison with the last available information from the German Federal Chamber of Pharmacies (ABDA) for the end of 2018 regarding the total number of CPs in Mecklenburg-Vorpommern ^37^ showed a 99% agreement. The minimum necessary sample size ( *n*) was determined for a population size (N) of 396 and an error margin ( *e*) of 0.05 using the following formula, which is based on a degree of variability of P=0.5 and a 95% confidence interval ^38^:

$$n=\frac{N}{1+N{\left(e\right)}^{2}}=\frac{396}{1+396{\left(0.05\right)}^{2}}=\frac{396}{1.99}=198.99$$

In Germany no studies have yet been conducted on a necessary referral to a doctor for acute diarrhoea by CPs. Therefore, the degree of variability is unknown. The assumed degree of variability of P=0.5 maximises the required sample size. The 396 CPs were stratified by location of the CP as an indicator for urban/rural and assigned a random number using the MS Excel random number generator and then simple random sampling was performed in each stratum to select the 199 participating CPs.

### Scenario and assessment

The Ordinance on the Operation of CPs in Germany includes an obligation for CPs to introduce a quality management system. The aim is to ensure a preferably adequate advice outcome ^39^. To ensure that this can be achieved, the Federal Chamber of Pharmacies has drafted various guidelines and tools, including the tool ‘Information and advice as part of self-medication using the example of self-diagnosis of diarrhoea’ ^40^. This forms the basis of the symptom-based scenario developed ( Table 1) and the evaluation form used. The recommendation to consult a doctor by pharmacy staff is defined as the appropriate outcome and the scenario was also designed according to this. The tool provided by the Federal Chamber of Pharmacies indicates 10 possible reasons (such as diarrhoea present > two to three days; fever > 39°C; blood or mucus in the stool; change from diarrhoea to constipation) that, if present, are considered to exceed the limits for self-medication and should result in the recommendation to consult a doctor. For this reason, a scenario was designed in which the grandmother’s diarrhoea had already been present for five days and she now had a fever of 40°C. Blood or mucus in the stool as another possible indication that the limits of self medication had been exceeded was not considered in the scenario because it did not seem realistic that a grandmother would share this intimate information with her grandchildren. Therefore, if the pharmacy staff asked the SPs about the presence of blood in the grandmother’s stool, the SPs stated that it was not known whether there was blood in the stool.

**Table 1.** Scenario.

Scenario
The SP enters the CP and asks for a medication for diarrhoea. The SP does not have a particular product in mind.
When questioned by the pharmacy staff, the following information is provided: - Preparation is for the 75-year-old grandmother - Diarrhoea present for five days - Not known how often the symptoms occur - Fever (40°C) since this morning; no vomiting; not known whether blood or mucus in the stool - No medical consultation to date - Existing medical conditions: Diabetes and high blood pressure; not known what medications are taken regularly

The evaluation form includes a total of eleven items with the first six items evaluating whether appropriate questions were asked. On this basis, the pharmacy staff should then decide whether to recommend that the patient consult a doctor (item seven). Because the tool also considers ‘dispensing a medication in an appropriate quantity up to consulting a doctor’ within the discretionary powers of the pharmacy staff, items eight to eleven evaluate whether a medication was dispensed and whether in the process information about the dosage, duration and side effects was provided also for the case of the limits of self-medication being exceeded and the necessary referral to consult a doctor.

Only objective items were used in order to avoid a subjective assessment and thus latitude in the evaluation by the SPs (for example, on the friendliness of the pharmacy staff). Therefore, only dichotomous scales were used (closed yes/no questions) to complete the individual items. To avoid the Hawthorne effect ^41^ and to ensure a realistic consultation situation, the visits took place without first informing the CPs included in the random sample in accordance with other national ^20, 21^ and international ^26, 42, 43^ studies.

### Data collection

As part of their three-semester research project at the Neubrandenburg University of Applied Sciences, two female and two male Masters students in the Health Sciences faculty, that is, four SPs, were available for the visits. Each CP was visited once (a total of 199 visits), whereby the CPs were distributed randomly across the four SPs (three SPs made 50 visits, one SP made 49 visits).

Before starting the data collection, each of the SPs familiarised themselves with the theoretical principles of the methodology and the contents of the evaluation form.

A pilot study with four visits was then carried out by each of the SPs to train the SPs in the use of the methodology and to verify the functionality of the collection form and the scenario. The total 16 visits were carried out in CPs that were not part of the stratified random sample. No changes to the scenario and the collection form were required after testing the scenarios.

The visits were carried out on different days of the week and at different times of the day. The SPs made their request to the pharmacy staff who first approached them. The SPs only provided additional information if they were then asked by the pharmacy staff to ensure that the information provided is invariable.

Along with the items on the evaluation form, the SPs planned, analogous to the international literature ( Table 2), to also collect a number of variables before, during and after the visits that may possibly affect the appropriate outcome.

**Table 2.** Possible influencing factors and time and type of data collection.

Possible influencing factors	Time of data collection	Type of data collection
Location of the CP ^14^ as indicator for urban/rural	Before the visit because stratification variable	Precise measurement by allocating the number of CPs identified to the particular location
CP quality certificate ^24^	After the visit	Precise measurement using a telephone query by the SP after completing the visits
Age of the pharmacy staff ^26^	During the visit	Estimate using visual impression of the SP
Gender of the pharmacy staff ^26^	During the visit	Exact measurement using visual impression of the SP
Professional group of the pharmacy staff ^27^	During and after the visit	Exact measurement based on the name tag, the receipt and, if necessary, using a telephone query by the SP after completing the visit
Queue – patients waiting after the SP ^44^	During the visit	Exact measurement using visual impression of the SP
Time of the visit ^25^	During the visit	Exact measurement using the SP’s watch
Number of questions asked ^25^	After the visit	Exact measurement by adding up the individual questions asked

Note: The possible influencing factors are taken from the relevant literature sources.

The corresponding evaluation form was completed immediately after the visits by the SPs to minimise any recall bias in the study results due to faulty memories. After termination of the study, each CP received general written performance feedback.

### Data analysis

Data were entered in duplicate into and analysed with SPSS software (IBM, Armonk, NY, USA), version 25 for Windows. As part of the descriptive statistics, frequencies and percentages were determined. Furthermore, 95% confidence intervals (CI) for categorical data using bootstrapping methods were also reported. Because the rule of thumb for sample size assumptions (minimum of 10 events per predictor variable) is given for a logistical regression ^45^, a binary logistic regression model was developed to identify the influence of various independent variables ( Table 2) on the appropriate outcome. All independent variables were checked for outliers and multicollinearity. According to Hosmer *et al.* ^46^, variables with a P value less than 0.25 in the bivariate analysis were included in the multivariate analysis. Crude odds ratios (COR) and adjusted odds ratios (AOR), 95% confidence intervals (CI) and P values were reported. A P value less than 0.05 was deemed significant in multivariate analysis.

### Ethical approval

The study protocol was approved by the institutional ethics committee of the Neubrandenburg University of Applied Sciences (registration number: HSNB/GPM/139/18). According to the ‘Guideline for the use of mystery research in market and social research’ ^47^, the data collected were anonymised and recorded in such a way that the CPs involved could not be identified. CPs were not asked for consent prior to the study being conducted because obtaining written consent would have significantly and negatively impacted the results. To resolve the issue of informed consent, the authors contacted the CPs by mail and email after the study informing them that an SP study had been conducted with the corresponding background information ^48, 49^. Recruited students provided their written informed consent to act as SPs.

## Results

All 199 planned visits were actually carried out, with a total of €784.70 used from the primary author’s own resources. Table 3 shows the socio-demographic data for the 199 CPs and the advising pharmacy staff.

**Table 3.** Socio-demographic data for the CPs and the advising pharmacy staff.

	Frequency (n)	Percentage (%)
All CPs	199	100
Location of the CP • 1 CP in the location • 2–4 CPs in the location • 5–19 CPs in the location • ≥ 20 CPs in the location	37 58 45 59	18.6 29.1 22.6 29.7
CP quality certificate • No • Yes • Not able to be determined	125 52 22	62.8 26.1 11.1
Age of the pharmacy staff • < 30 • 30–49 • ≥ 50	39 110 50	19.6 55.3 25.1
Gender of the pharmacy staff • Male • Female	20 179	10.1 89.9
Professional group of the pharmacy staff • Pharmacist • Non-pharmacist • Not able to be determined	54 90 55	27.1 45.2 27.7

This reveals that the CPs are mostly in locations with competing CPs nearby. In addition, only a minority of the CPs had a quality certificate. In most of the visits, the advising pharmacy staff were female, between 30 and 49 years of age and were non-pharmacists, i.e. pharmacy technicians and pharmaceutical technical assistants.

The appropriate outcome was achieved in 59.8% (n=119) of all visits ( Table 4). The question ‘for whom is the medication?’ was most asked (75.4%, n=150), while ‘clarification by a doctor’ was asked the least (17.6%, n=35). In 87.9% (n=175) of all visits a medication was dispensed, whereas in 22.1% (n=24) of all visits no medication was dispensed. Regarding the visits in which a medication was dispensed, in 77.2% (n=135) of visits loperamide was dispensed and in 11.4% (n=20) probiotics were dispensed, whereas, for example, antibiotics that require a prescription in Germany were not dispensed at all. In 88.6% (n=155) information was provided about the dosage of the medication, whereas information about possible side effects was only provided by the pharmacy staff in 8.0% (n=14) of visits.

**Table 4.** Assessment items on the evaluation form (n = 199).

	Yes		
	Frequency (n)	Percentage (%)	95% CI
1. For whom is the medication?	150	75.4	69.3–81.4
2. How long have the symptoms been present?	97	48.7	42,2–55.8
3. How often do the symptoms occur?	46	23.1	17.6–29.1
4. Have other symptoms occurred?	56	28.1	22.1–34.7
5. Have the symptoms already been clarified by a doctor?	35	17.6	12.6–23.1
6. Are there other medical conditions or which medications are taken regularly?	65	32.7	26.1–39.2
7. Is a medical consultation recommended (appropriate outcome)?	119	59.8	53.3–66.3
8. Dispensing of a medication	175	87.9	83.4–92.5
9. Information about dosage	155	88.6	83.4–93.1
10. Information about duration	82	46.9	39.4–54.3
11. Information about side effects	14	8.0	4.0–12.6

The binary logistic regression model is shown in Table 5. Bivariate analysis demonstrated that only three (quality certificate, professional group of the pharmacy staff, number of questions asked) of eight predictor variables having a P value < 0.25 were included in a multivariate logistic regression model.

**Table 5.** Possible factors influencing the recommendation of a necessary medical consultation.

Possible influencing factors and categories	Total n = 199 n (%)	Referral n = 119 n (%)	No referral n = 80 n (%)	COR (95% CI)	P value	AOR (95% CI)	P value
Location of the CP • 1 CP in the location • 2–4 CPs in the location • 5–19 CPs in the location • ≥ 20 CPs in the location	37 (18.6) 58 (29.1) 45 (22.6) 59 (29.7)	24 (20.2) 33 (27.7) 27 (22.7) 35 (29.4)	13 (16.2) 25 (31.3) 18 (22.5) 24 (30.0)	1 0.715 (0.305–1.676) 0.813 (0.330–2.000) 0.790 (0.337–1.851)	0.440 0.651 0.587		
CP quality certificate • No • Yes • Not able to be determined	125 (62.8) 52 (26.1) 22 (11.1)	70 (58.8) 35 (29.4) 14 (11.8)	55 (68.8) 17 (21.2) 8 (10.0)	1 1.618 (0.821–3.188) 1.375 (0.538–3.512)	0.165 0.506		
Age of the pharmacy staff • < 30 • 30–49 • ≥ 50	39 (19.6) 110 (55.3) 50 (25.1)	21 (17.6) 66 (55.5) 32 (26.9)	18 (22.5) 44 (55.0) 18 (22.5)	1 1.286 (0.616–2.684) 1.524 (0.649–3.580)	0.503 0.334		
Gender of the pharmacy staff • Male • Female	20 (10.1) 179 (89.9)	10 (8.4) 109 (91.6)	10 (12.5) 70 (87.5)	1 1.557 (0.617–3.933)	0.349		
Professional group of the pharmacy staff • Pharmacist • Non-pharmacist • Not able to be determined	54 (27.1) 90 (45.2) 55 (27.7)	25 (21.0) 54 (45.4) 40 (33.6)	29 (36.3) 36 (45.0) 15 (18.7)	1 1.740 (0.880–3.439) 3.093 (1.391–6.877)	0.111 0.006	1 2.446 (1.025–5.835) 3.269 (1.208–8.843)	0.044 * 0.020 *
Time of the visit • 8:00 am – 12:00 pm • 12:01 pm – 4:00 pm • 4:01 pm – 8:00 pm	77 (38.7) 89 (44.7) 33 (16.6)	48 (40.3) 49 (41.2) 22 (18.5)	29 (36.3) 40 (50.0) 11 (13.7)	1 0.740 (0.397–1.379) 1.208 (0.512–2.850)	0.343 0.666		
Queue • No • Yes	146 (73.4) 53 (26.6)	86 (72.3) 33 (27.7)	60 (75.0) 20 (25.0)	1 1.151 (0.603–2.196)	0.669		
Number of questions asked • None • one • two • ≥ three	36 (18.1) 39 (19.6) 41 (20.6) 83 (41.7)	7 (5.9) 10 (8.4) 32 (26.9) 70 (58.8)	29 (36.3) 29 (36.3) 9 (11.2) 13 (16.2)	1 1.429 (0.478–4.268) 14.730 (4.863–44.616) 22.308 (8.079–61.597)	0.523 < 0.001 < 0.001	1 15.291 (4.876–47.955) 23.406 (8.204–66.773)	<0.001 * <0.001 *

Abbreviations: COR = Crude Odds Ratio; AOR = Adjusted Odds Ratio. * significant at P < 0.05.

In this multivariate analysis, being a non-pharmacist (AOR = 2.446; 95% CI = 1.025–5.835; p = 0.044) or pharmacy staff with an undetermined professional group (AOR = 3.269; 95% CI = 1.208–8.843; p = 0.020) as opposed to a pharmacist and two (AOR = 15.291; 95% CI = 4.876–47.955; p < 0.001) or three or more (AOR = 23.406; 95% CI = 8.204–66.773; p < 0.001) questions being asked as opposed to no questioning were two factors that were significantly associated with referral to a doctor. The location of the CP, the presence of a quality certificate and a queue, the age and the gender of the pharmacy staff as well as the time of day of the visits did not have any significant effect on the appropriate outcome. The model returned a Nagelkerke R ^2^ value of 0.45.

## Discussion

Although the appropriate outcome, that is, the recommendation to consult a doctor, was achieved in approximately 60% of all visits, approximately 40% of all CPs did not recommend that the patient consult a doctor even though this would have been necessary. Due to the design of the scenario as a symptom-based query, the study results obtained would probably be worse in everyday consultations with its mix of symptom- and product-based queries. This is reflected in the international and national literature in which (otherwise identical) scenarios showed a better quality of advice for a symptom-based query than for a medication-based query ^20, 50^. In another study a recommendation rate for a necessary medical consultation of 57% was determined for a medication-based query, likewise for acute diarrhoea in adults, in Australian CPs ^27^. Considerably worse results for the recommendation of a necessary medical consultation are seen in the international literature for other indications such as pain (recommendation rate: approx. 27% ^43^), vaginal thrush (recommendation rates: 10% and 22% ^51^) and asthma (recommendation rates: 40% and 19% ^52^), which were all medication-based queries. In contrast, the only study that has investigated the recommendation for a necessary medical consultation to date in Germany showed a very high recommendation rate of 90%, again for a medication-based query for an antacid ^13^.

Possible reasons discussed in the international literature for the poor quality of advice provided include lack of time, manpower, interest and knowledge on behalf of the pharmacy staff ^10, 50, 53, 54^. In regards to a lack of knowledge, pharmacists should receive greater training during their studies in patient consultations using examples ^44^, especially as to date the teaching of such ‘soft skills’ in German universities has been below average compared to other European countries ^55^. In addition, the systematic use of checklists could also help to ensure a better quality of advice in daily patient contact ^56^.

On the other hand, the international literature reveals that pharmacy staff most definitely have the knowledge to provide good advice, but it is only inadequately applied in actual patient contact ^53, 57– 59^. In a German study, pharmacy staff suggested that this is due to the worry that the client may feel patronised ^13^. In contrast to this, another German study determined that most patients would like to have a consultation in the CP; however, the study design meant that rather health-conscious and therefore not representative study participants were included ^60^. In a very recent German study, it was stated by pharmacy staff that patients frequently did not want any advice in terms of drug handling, sometimes due to a lack of time ^61^. In a somewhat older English study, 62.5% of the patients interviewed did not expect to be asked any questions by the pharmacy staff during their last purchase of a non-prescription medication ^62^. Similar results were obtained in a more recent study in Qatar in which the pharmacist cited ‘no interest by the patients’ as the central reason for providing insufficient advice ^42^. Another very recent qualitative study from Australia was also able to determine in this context that from the perspective of both the consumers and the pharmacy staff, the frequent lack of privacy is also an important reason for the poor exchange of information ^63^. It would, therefore, be necessary to require CP owners in future to provide appropriate spaces that ensure privacy.

Analogous to other international studies ^52, 64, 65^, the number of questions asked had a significant effect on the appropriate outcome. This is based on the rationale that the right decision – in our case to recommend consulting a doctor – can only be made if appropriate questions are asked and thus information is obtained about the patient. In contrast to this, a Saudi Arabian study revealed that there was no relationship between the number of questions asked and the appropriate outcome – in this case, the dispensing of prescription medications without a medical prescription ^25^. However, the outcome ‘dispensing prescription medications without a medical prescription’ is not comparable to the outcome ‘recommendation to consult a doctor’. While for the first a sensible decision can be made even without asking appropriate questions (that is, refusing to dispense a prescription medication without a corresponding medical prescription), for the latter a sensible decision is only possible on the basis of appropriately asked questions.

In regards to individual questions, the question about the duration of the acute diarrhoea was asked more frequently compared to similar national studies ^20, 21^. In a recent SP study, again for acute diarrhoea in adults, that was conducted in Iraq, this question was asked in almost 80% of all visits ^66^. In contrast, the results are considerably worse for a comparable Turkish SP study which also investigated the quality of advice provided for acute diarrhoea in adults in which this question was only asked in 26% of all visits ^67^. Because certain medications can cause diarrhoea, asking about any pre-existing medical conditions or the patient’s medical history is particularly important ^68^. This question was asked in this study in approximately one-third of the visits and is thus more frequent than in the comparable national studies ^20, 21^. In the similar Turkish SP study, this question was not asked in a single visit ^67^.

Depending on the criteria being investigated, there are also clear differences in the information that is given to the patients when medication is dispensed. Analogous to the national ^20, 21^ and international literature ^57, 69^, information is often provided about the dosage of the medication but rarely about possible side effects. An active query by patients could help to greatly improve the frequency of advice provided for the criterion ‘side effects’, as was shown in a recent SP study from Tanzania ^70^.

Along with the number of questions asked, the professional group of the pharmacy staff providing the advice also had a significant influence on the appropriate outcome. Non pharmacists (pharmaceutical technical assistants and pharmacy technicians) have a significantly higher likelihood of achieving the appropriate outcome than pharmacists. On the other hand, two comparable SP studies from Germany and Australia – also for acute diarrhoea in adults – did not show any significant difference in the quality of advice provided by the two professional groups ^21^ or a significantly higher likelihood of pharmacists ^27^ achieving the appropriate outcome. In the Turkish SP study pharmacists made significantly more recommendations to their patients than pharmaceutical technical assistants ^67^. For indications other than acute diarrhoea in adults there was an analogous picture – either a significantly better quality of advice provided by pharmacists compared to non-pharmacists ^14, 65^ or no significant differences between the two professional groups ^50, 57, 71^. Due to inconsistent national and international studies and given the surprising results obtained in this study, future studies should investigate in greater depth this influencing factor for the advice provided.

### Strengths and limitations

This is the first German study that investigated to what degree CPs recommend a necessary medical consultation for acute diarrhoea in adults. The sample size used in the study (n=199) is also considerably larger than the international median sample size (n=112) for SP studies ^72^. An SP study design also avoids social desirability, unlike a survey. By using 4 SPs, the study also satisfies the requirement of not having fewer than 2 (generalisable) but also not too many (standardisable) SPs ^72^.

Conducting the pilot study with persons other than the researchers may have improved the approach ^73^, because the perspective of persons not involved in developing the study design would have been considered. On the other hand, carrying out the pilot study also aimed to train the SPs in the use of the methodology, which again would no longer have been possible. By way of qualification, it must be stated that the results only refer to one scenario. In addition, only one indication was investigated. Because the study results only refer to one German state, future studies should be expanded to include additional or all German states. The results also refer only to a quite specific time because the study is a cross-sectional study. There is further need for research to determine whether and, if yes, how the results change over time, which is only possible with a longitudinal study design. Carrying out the visits in winter only may have had a seasonal impact on the findings because the pharmacy staff may possibly have been more aware of this indication due to the increased occurrence of acute diarrhoea in the winter months and therefore the results of our study may have been better than at other times of the year ^74^. The audio recordings recommended in the literature for quality assurance ^75^ must be omitted for data privacy reasons because all CPs would have to be informed about this in advance, which would jeopardise the covert study design. Although the particular evaluation forms were filled out by the SPs immediately after visiting the CPs and only objective evaluation criteria were used, recall bias ^26^ and the intra- and inter-observer variabilities typical for SP studies ^76^ cannot be completely ruled out. For greater quality assurance, future SP studies could always carry out and evaluate visits in parallel using two persons (1 SP and 1 observer) ^77^. Although in the literature it is recommended for reasons of effectiveness to provide performance feedback to initiate improvement measures immediately after the particular visit ^23^, this was omitted because the student SPs would probably not be accepted by pharmacy staff as briefing partners ^14^. The restrictions on time and resources and the very high number of CPs visited mean that it was also not possible to provide the CPs with individual written performance feedback including benchmarking after the visits.

### Study recommendations

The findings of this study recommend further research to identify why the advice provided is so often poor and particularly why the quality of advice provided by pharmacists is so inadequate. The results should make both the Mecklenburg Vorpommern pharmacy association and legislators aware of the need to significantly escalate their quality management efforts. In this context appropriate mandatory continuing education courses as well as regular independent reviews with an adequate sanction mechanism could provide a stimulus to sustainably improve the quality of advice ^78, 79^.

## Conclusions

In almost half of the visits a necessary referral to a doctor was not recommended. Furthermore, the quality of questioning and the quality of information were below expectations. Moreover, two or more questions and, surprisingly, the involvement of non pharmacists were identified as relevant factors influencing the appropriate outcome.

## Data availability

### Underlying data

Harvard Dataverse: Replication Data for: Do north-eastern German pharmacies recommend a necessary medical consultation for acute diarrhoea? Magnitude and determinants using a simulated patient approach, https://doi.org/10.7910/DVN/5KVLW4 ^80^

Data are available under the terms of the Creative Commons Zero “No rights reserved” data waiver (CC0 1.0 Public domain dedication).
