# Supplementary material for: Do north-eastern German pharmacies recommend a necessary medical consultation for acute diarrhoea? Magnitude and determinants using a simulated patient approach
Source: F1000Res. 2020 May 7;8:1841. Originally published 2019 Nov 1. [Version 2] doi: 10.12688/f1000research.21045.2 (PMC7014574; doi:10.12688/f1000research.21045.2)
Supplement: Peer review history

# Peer review: Reviewer response for version 2

**DOI:** 10.5256/f1000research.26136.r63148

**Published:** 2020-06-09

**Authors:** Subish Palaian

**Affiliations:** 1 Department of Clinical Sciences, College of Pharmacy and Health Sciences, Ajman University, Ajman, United Arab Emirates

Is the work clearly and accurately presented and does it cite the current literature?

Yes

If applicable, is the statistical analysis and its interpretation appropriate?

I cannot comment. A qualified statistician is required.

Are all the source data underlying the results available to ensure full reproducibility?

Yes

Is the study design appropriate and is the work technically sound?

Yes

Are the conclusions drawn adequately supported by the results?

Partly

Are sufficient details of methods and analysis provided to allow replication by others?

Yes

Reviewer Expertise:

NA

I confirm that I have read this submission and believe that I have an appropriate level of expertise to confirm that it is of an acceptable scientific standard.

# Peer review: Reviewer response for version 2

**DOI:** 10.5256/f1000research.26136.r63149

**Published:** 2020-05-21

**Authors:** Mohamed Izham Mohamed Ibrahim

**Affiliations:** 1 Department of Clinical Pharmacy and Practice, College of Pharmacy, Qatar University, Doha, Qatar

I have reviewed the responses and changes made in the revised manuscript. I am satisfied with the improvement.  With the present quality, I approve this manuscript for indexing.

Is the work clearly and accurately presented and does it cite the current literature?

Yes

If applicable, is the statistical analysis and its interpretation appropriate?

Yes

Are all the source data underlying the results available to ensure full reproducibility?

Yes

Is the study design appropriate and is the work technically sound?

Yes

Are the conclusions drawn adequately supported by the results?

No

Are sufficient details of methods and analysis provided to allow replication by others?

Yes

Reviewer Expertise:

NA

I confirm that I have read this submission and believe that I have an appropriate level of expertise to confirm that it is of an acceptable scientific standard.

# Peer review: Reviewer response for version 1

**DOI:** 10.5256/f1000research.23162.r58282

**Published:** 2020-02-11

**Authors:** Subish Palaian

**Affiliations:** 1 Department of Clinical Sciences, College of Pharmacy and Health Sciences, Ajman University, Ajman, United Arab Emirates

I enjoyed reading the manuscript. It is a well conducted study with robust methodology and focusing on ‘community pharmacist referral’ which is an important aspect of community pharmacy practice. I thank the F1000Research for providing me the opportunity in reviewing this manuscript. The ‘simulated client method’ used in this research is suitable in evaluating the real-life practice happening at the community pharmacies.

 I have few concerns associated with the manuscript and are as below:

**Abstract:**

  

- The result section of the abstract mentions about ‘non-pharmacist’. It would be nice to elaborate more on those non-pharmacists somewhere in the Introduction section. Though it is mentioned in the Results, para 2, one would like to know whether non-pharmacists are allowed to dispense medications. This may be useful for international readers. Similarly, in the result part the statement ‘two or more questions as opposed to no questions were significantly associated with a referral to a doctor’ doesn’t appear to be novel. It is understood that someone who had not asked any question will be unable to refer. Authors might consider other observations to be mentioned at this section of the abstract.

 

**Introduction:**

  

- The paragraph 6, authors need to clearly mention which ‘baseline’ and ‘follow-up’ study are they mentioning. What is the relation between the current study and those previous studies. Are they the same sample?
- I also found some similarity with few of the previous publications by the author using the same 'acute diarrhea' scenario. Authors need to verify the contents and avoid any similarities. 
- May be the Introduction section should elaborate more on the justification of this research.  Why did all these studies used only ‘acute diarrhea’ as the scenario? This needs further elaboration.

 

**Methodology**

- Under scenario and assessment, it is not clear how authors categorized ‘Diarrhoea being present for five days as well as a high fever (40°C) are both considered to exceed the limits of self-medication’. I suppose even if there is presence of mucus and/or blood in the stool may be considered referral. I suggest more explanation on development of the scenario and content validation and why ‘Dysentery’ was excluded from the scenario.  

 

**Results:**

  

- In this research, 45.2% of the dispensers were ‘non-pharmacists’. If that is the case then how can we correlate the research findings to evaluate the Community Pharmacy practice? The term ‘non-pharmacist’ needs more elaboration. Probably in the Introduction section, as I mentioned elsewhere.
- Table 4, response 8. Dispensing of a medication 175 (87.9%). What were these medications. Were there any antibiotics? What happened to the rest 22.1%? Why they did not dispense medications? These are some missing information and needs to be incorporated in the Results section.   
  ​​​

**Discussion:**

- The discussion can focus on reason for non-referral by the pharmacists. Are there skills related issues? Do the pharmacists possess adequate skills in assessing patients with diarrhea? These aspects may further enrich the discussion.

 

**Limitations**:

- Authors could explore any seasonal impact on the finding.

Is the work clearly and accurately presented and does it cite the current literature?

Yes

If applicable, is the statistical analysis and its interpretation appropriate?

I cannot comment. A qualified statistician is required.

Are all the source data underlying the results available to ensure full reproducibility?

Yes

Is the study design appropriate and is the work technically sound?

Yes

Are the conclusions drawn adequately supported by the results?

Partly

Are sufficient details of methods and analysis provided to allow replication by others?

Yes

Reviewer Expertise:

Pharmacy Practice, Pharmacovigilance, Pharmacoepidemiology

I confirm that I have read this submission and believe that I have an appropriate level of expertise to confirm that it is of an acceptable scientific standard, however I have significant reservations, as outlined above.

# Response

**Published:** 2020-04-24

**Authors:** Bernhard Langer

**Affiliations:** University of Applied Sciences Neubrandenburg, Germany

**We would like to thank the reviewer for taking the time to review our manuscript and also for the very helpful and detailed comments, recommendations and questions. See our point-by-point response to the reviewer’s comments below.**

I enjoyed reading the manuscript. It is a well conducted study with robust methodology and focusing on ‘community pharmacist referral’ which is an important aspect of community pharmacy practice. I thank the F1000Research for providing me the opportunity in reviewing this manuscript. The ‘simulated client method’ used in this research is suitable in evaluating the real-life practice happening at the community pharmacies.

I have few concerns associated with the manuscript and are as below:

Abstract:

- The result section of the abstract mentions about ‘non-pharmacist’. It would be nice to elaborate more on those non-pharmacists somewhere in the Introduction section. Though it is mentioned in the Results, para 2, one would like to know whether non-pharmacists are allowed to dispense medications. This may be useful for international readers.

 

**Thank you for this comment. The following sentence has been added to the background section of the abstract: ‘In Germany, non-pharmacists (pharmacy technicians and pharmaceutical technical assistants) are permitted to advise on and sell medications in addition to pharmacists.’**


** **

- Similarly, in the result part the statement ‘two or more questions as opposed to no questions were significantly associated with a referral to a doctor’ doesn’t appear to be novel. It is understood that someone who had not asked any question will be unable to refer. Authors might consider other observations to be mentioned at this section of the abstract.

 

**Thank you for this comment. We replaced the statement ‘two or more questions as opposed to no questions were significantly associated with a referral to a doctor’ with other observations.**

Introduction:

 

- The paragraph 6, authors need to clearly mention which ‘baseline’ and ‘follow-up’ study are they mentioning. What is the relation between the current study and those previous studies. Are they the same sample?

 

**Thank you for this comment. We expressed ourselves misleadingly.To clarify, the sentence has been restructured as follows: ‘Despite the two studies from 2014 and 2017 mentioned above, there remains a lack of information about the quality of advice for the indication acute diarrhoea in Germany. The reason is that the four scenarios investigated in these two studies were designed as ‘moderate’, in that referral to a medical consultation by the pharmacy staff was not mandatory in any of the scenarios.’ Also to clarify, the word ‘current’ has been added to this sentence: ‘The aim of this current study was, therefore, to determine on the basis of a considerably larger number of pharmacies whether the pharmacy staff referred patients to a medical consultation for a scenario in which consulting a doctor was mandatory (‘appropriate outcome’) and what the quality of questioning and – if a medication was dispensed – the quality of information provided were in this context.’ The following sentence was also added to the design subsection in the methods section: ‘It must once more be explicitly emphasized that this study shares no similarities with the previous studies conducted in 2014 and 2017 apart from the indication investigated (acute diarrhoea).’**

 

- I also found some similarity with few of the previous publications by the author using the same 'acute diarrhea' scenario. Authors need to verify the contents and avoid any similarities.

 

**Thank you for this comment. This study and the previous studies investigate the same indication (acute diarrhoea) but do not use the same scenario. As described in the introduction section, the scenarios used in previous studies from 2014 and 2017 were designed as ‘moderate’ in that in none of the scenarios was a referral for medical consultation by the pharmacy staff mandatory. In contrast, this study determines whether the pharmacy staff referred patients to a medical consultation in a scenario in which consulting a doctor was mandatory. Therefore, it is simulated – unlike the studies from 2014 and 2017 – that the grandmother has had diarrhoea for five days and fever (40°C) (see Table 1). To clarify, the following sentence has been added to the design subsection in the methods section: ‘It must once more be explicitly emphasized that this study shares no similarities with the previous studies conducted in 2014 and 2017 apart from the indication investigated (acute diarrhoea).’**

 

- May be the Introduction section should elaborate more on the justification of this research.  Why did all these studies used only ‘acute diarrhea’ as the scenario? This needs further elaboration.

 

**Thank you for this comment. As mentioned in the introduction section, there are studies available from Germany regarding the quality of advice provided in pharmacies but none for this indication. To better clarify why three research studies for acute diarrhoea are necessary, the sentences have been restructured as follows: ‘Because of this lack of studies for acute diarrhoea, the quality of advice provided for acute diarrhoea in adults was analysed for Germany for the first time in 2014 and also identified clear deficiencies. Despite performance feedback used in the process to improve the quality of advice, a follow-up study in 2017 identical to that conducted in 2014 again showed poor quality of advice.’ And furthermore: ‘Despite the two studies from 2014 and 2017 mentioned above, there remains a lack of information about the quality of advice for the indication acute diarrhoea in Germany. The reason is that the four scenarios investigated in these two studies were designed as “moderate”, in that referral to a medical consultation by the pharmacy staff was not mandatory in any of the scenarios.’**

Methodology

 

- Under scenario and assessment, it is not clear how authors categorized ‘Diarrhoea being present for five days as well as a high fever (40°C) are both considered to exceed the limits of self-medication’. I suppose even if there is presence of mucus and/or blood in the stool may be considered referral. I suggest more explanation on development of the scenario and content validation and why ‘Dysentery’ was excluded from the scenario.

 

**Thank you for this comment. More explanation on development of the scenario and content validation has been given. Additionally, it has been explained why ‘Dysentery’ was excluded from the scenario. Therefore, the relevant sentences have been restructured as follows: ‘The tool provided by the Federal Chamber of Pharmacies indicates 10 possible reasons (such as diarrhoea present > two to three days; fever > 39°C; blood or mucus in the stool; change from diarrhoea to constipation) each of which, if present, are considered to exceed the limits for self-medication and should result in the recommendation to consult a doctor. For this reason, a scenario was designed in which the grandmother’s diarrhoea had already been present for five days and she also had a fever of 40°C. Blood or mucus in the stool as another possible indication that the limits of self-medication had been exceeded was not considered in the scenario because it did not appear realistic for a grandmother to share this intimate information with her grandchildren. Therefore, if the pharmacy staff asked the SPs about the presence of blood in the grandmother’s stool, the SPs stated that it was not known whether there was blood in the stool.’**

Results:

 

- In this research, 45.2% of the dispensers were ‘non-pharmacists’. If that is the case then how can we correlate the research findings to evaluate the Community Pharmacy practice? The term ‘non-pharmacist’ needs more elaboration. Probably in the Introduction section, as I mentioned elsewhere.

 

**Thank you for this comment. The following sentence has been added to the introduction section to better explain the term ‘non-pharmacist’: “In Germany, non-pharmacists (pharmacy technicians and pharmaceutical technical assistants) are permitted to advise on and sell medications in addition to pharmacists.’**

 

- Table 4, response 8. Dispensing of a medication 175 (87.9%). What were these medications. Were there any antibiotics? What happened to the rest 22.1%? Why they did not dispense medications? These are some missing information and needs to be incorporated in the Results section.

 

**Thank you for this comment. The relevant sentences have been restructured as follows: ‘In 87.9% (n=175) of all visits a medication was dispensed, whereas in 22.1% (n=24) of all visits no medication was dispensed. Regarding the visits in which a medication was dispensed, in 77.2% (n=135) of visits loperamide was dispensed and in 11.4% (n=20) probiotics were dispensed, whereas, for example, antibiotics that require a prescription in Germany were not dispensed at all.’ As described previously in the methods section, the guidelines expect that for such a condition that pharmacists are free to determine whether they also dispense a medication in addition to a mandatory referral to a doctor. It is therefore no wonder that a medication was not dispensed in all test purchases.**

Discussion:

 

- The discussion can focus on reason for non-referral by the pharmacists. Are there skills related issues? Do the pharmacists possess adequate skills in assessing patients with diarrhea? These aspects may further enrich the discussion.

 

**Thank you for this comment. To further enrich the discussion, the following sentence and an additional reference has been added: ‘In regards to a lack of knowledge, pharmacists should receive greater training during their studies in patient consultations using examples ^44 ^, especially as to date the teaching of such ‘soft skills’ in German universities has been below average compared to other European countries ^55^ .’**

Limitations:

 

- Authors could explore any seasonal impact on the finding.

 

**Thanks a lot for this comment. The following sentence and an additional reference to the limitations have been added: ‘Carrying out the visits in winter only may have had a seasonal impact on the findings because the pharmacy staff may possibly have been more aware of this indication due to the increased occurrence of acute diarrhoea in the winter months and therefore the results of our study may have been better than at other times of the year ^74^ .’**

# Peer review: Reviewer response for version 1

**DOI:** 10.5256/f1000research.23162.r59628

**Published:** 2020-02-10

**Authors:** Suleiman I. Sharif

**Affiliations:** 1 Department of Pharmacy Practice and Pharmacotherapeutics, College of Pharmacy, University of Sharjah, Sharjah, United Arab Emirates

I thank you for trusting me with the review of this article which presents a well-designed and executed study. I do approve of indexing the article after some answers and minor corrections to be provided by the authors.

1. Ethical approval: I appreciate that in this study obtaining a written consent from the staff of the pharmacies would have greatly and negatively impacted the results and any “appropriate outcome” obtained would have been a biased one.
2. Pre-piloting the scenario with other than the researchers may have improved the approach.
3. The SPs made their request to the pharmacy staff who first approached them. Were the SPs certain whether “the pharmacy staff who first approached them” a pharmacist or pharmacy technician? In table 3, about 72% were not pharmacist and not able to determine. Moreover, the authors stated in the results that “In most of the test purchases, the advising pharmacy staff were females, between 30 and 49 years of age and did not have a pharmacy degree (non-pharmacist)”. Since those they should practice under the supervision of a pharmacist, the authors need to elaborate on this in relation to their discussion where they state” Non-pharmacists (pharmaceutical technical assistants and pharmacy technicians) have a significantly higher likelihood of achieving the appropriate outcome than pharmacists”.
4. Point 2 must be also discussed in relation to their conclusion where they state” Furthermore, more questioning and involvement by non-pharmacists was identified as relevant factors influencing the appropriate outcome. There is a further need for research to identify why the advice provided is so often poor and particularly why the quality of advice provided by pharmacists is so inadequate”.
5. In table 5, under Queue and Number of questions asked the percentages do not total to 100%. Therefore, the authors need to check their original data, or declare “Missing data” in the table to total the percentage to 100%.

Is the work clearly and accurately presented and does it cite the current literature?

Yes

If applicable, is the statistical analysis and its interpretation appropriate?

Yes

Are all the source data underlying the results available to ensure full reproducibility?

No source data required

Is the study design appropriate and is the work technically sound?

Yes

Are the conclusions drawn adequately supported by the results?

Yes

Are sufficient details of methods and analysis provided to allow replication by others?

Yes

Reviewer Expertise:

Drug Utilization Studies, Pharmacy education, Pharmacy Practice , Neuropharmacology

I confirm that I have read this submission and believe that I have an appropriate level of expertise to confirm that it is of an acceptable scientific standard.

# Response

**Published:** 2020-04-24

**Authors:** Bernhard Langer

**Affiliations:** University of Applied Sciences Neubrandenburg, Germany

**We would like to thank the reviewer for taking the time to review our manuscript and also for the very helpful and detailed comments, recommendations and questions. See our point-by-point response to the reviewer’s comments below.**

I thank you for trusting me with the review of this article which presents a well-designed and executed study. I do approve of indexing the article after some answers and minor corrections to be provided by the authors.

 

- Ethical approval: I appreciate that in this study obtaining a written consent from the staff of the pharmacies would have greatly and negatively impacted the results and any “appropriate outcome” obtained would have been a biased one.

 

**Thank you for this comment. Yes, that is why we did not ask for consent to participate in advance. To clarify, the relevant sentence has been restructured as follows: ‘CPs were not asked for consent to participate prior to the study being conducted because obtaining written consent would have significantly and negatively impacted the results.’ Additionally, the following sentence was added: ‘To resolve the issue of informed consent, the authors contacted the CPs by mail and email after the study informing them that an SP study had been conducted with the corresponding background information ^48, 49^ .’**

 

- Pre-piloting the scenario with other than the researchers may have improved the approach.

**Thank you for this comment. Therefore, the following sentences and an additional reference have been added to the limitations: ‘Conducting the pilot study with persons other than the researchers may have improved the approach ^73^ , because the perspective of persons not involved in developing the study design would have been considered. On the other hand, carrying out the pilot study also aimed to train the SPs in the use of the methodology, which again would no longer have been possible.’**

 

- The SPs made their request to the pharmacy staff who first approached them. Were the SPs certain whether “the pharmacy staff who first approached them” a pharmacist or pharmacy technician?

**Thank you for this comment. This information was given in Table 2: ‘Exact measurement based on the name tag, the receipt and, if necessary, using a telephone query by the SP after completing the test purchase.’ Despite these efforts, the professional group of the pharmacy staff could not be determined in 55 of 199 test purchases.**

 

- In table 3, about 72% were not pharmacist and not able to determine. Moreover, the authors stated in the results that “In most of the test purchases, the advising pharmacy staff were females, between 30 and 49 years of age and did not have a pharmacy degree (non-pharmacist)”. Since those they should practice under the supervision of a pharmacist, the authors need to elaborate on this in relation to their discussion where they state **‘**Non-pharmacists (pharmaceutical technical assistants and pharmacy technicians) have a significantly higher likelihood of achieving the appropriate outcome than pharmacists. **’**

 

**Thank you for this comment. We have expressed ourselves misleadingly. Therefore, we have deleted ‘but only under the supervision of pharmacists’ in the introduction. To better explain the word ‘non-pharmacist’, we have additionally restructured the sentence as follows: ‘In Germany, non-pharmacists (pharmacy technicians and pharmaceutical technical assistants) are permitted to advise on and sell medications in addition to pharmacists.’ Therefore, we believe that the sentence ‘Non-pharmacists (pharmaceutical technical assistants and pharmacy technicians) have a significantly higher likelihood of achieving the appropriate outcome than pharmacists.’ in the discussion section is now clearer.**

 

- Point 2 must be also discussed in relation to their conclusion where they state “Furthermore, more questioning and involvement by non-pharmacists was identified as relevant factors influencing the appropriate outcome. There is a further need for research to identify why the advice provided is so often poor and particularly why the quality of advice provided by pharmacists is so inadequate”.

 

**Thank you for this comment. Please see our remarks to your point 3. We have deleted ‘but only under the supervision of pharmacists’ and have restructured the relevant sentence in the introduction section as follows: ‘In Germany, non-pharmacists (pharmacy technicians and pharmaceutical technical assistants) are permitted to advise on and sell medications in addition to pharmacists.’ Therefore, we believe that the sentences ‘Moreover, two or more questions and, surprisingly, the involvement of non-pharmacists were identified as relevant factors influencing the appropriate outcome.’ in the conclusions and ‘The findings of this study recommend further research to identify why the advice provided is so often poor and particularly why the quality of advice provided by pharmacists is so inadequate.’ in the study recommendations is easier to understand.**

 

- In table 5, under Queue and Number of questions asked the percentages do not total to 100%. Therefore, the authors need to check their original data, or declare “Missing data” in the table to total the percentage to 100%.

 

**Thank you for this comment. We have checked all percentages reported in tables and made corrections if necessary.**

# Peer review: Reviewer response for version 1

**DOI:** 10.5256/f1000research.23162.r56075

**Published:** 2019-11-15

**Authors:** Mohamed Izham Mohamed Ibrahim

**Affiliations:** 1 Department of Clinical Pharmacy and Practice, College of Pharmacy, Qatar University, Doha, Qatar

Thank you for allowing me to review this article.  It is an interesting study using an interesting approach i.e. SP.  I have a few comments:

1. The conclusion in the abstract section must be consistent with the objective of the study and main findings.
2. Percentages reported in tables must equal to 100% - kindly check all figures.
3. Tables cited in the main text should in proper order.
4. Even though due to the nature of the study, subjects selected were not asked for consent, did the authors contact them after the study informing that a study was conducted with reasons? This is one way that the researchers could resolve the issue of informed consent prior to the study.
5. Create a paragraph on Study recommendations at the end of the Discussion section.  Consider to move some parts of the conclusion. Rewrite the conclusion section.

Is the work clearly and accurately presented and does it cite the current literature?

Yes

If applicable, is the statistical analysis and its interpretation appropriate?

Yes

Are all the source data underlying the results available to ensure full reproducibility?

Yes

Is the study design appropriate and is the work technically sound?

Yes

Are the conclusions drawn adequately supported by the results?

No

Are sufficient details of methods and analysis provided to allow replication by others?

Yes

Reviewer Expertise:

Social-behavioral and Administrative aspects of health and pharmacy

I confirm that I have read this submission and believe that I have an appropriate level of expertise to confirm that it is of an acceptable scientific standard, however I have significant reservations, as outlined above.

# Response

**Published:** 2020-04-24

**Authors:** Bernhard Langer

**Affiliations:** University of Applied Sciences Neubrandenburg, Germany

**We would like to thank the reviewer for taking the time to review our manuscript and also for the very helpful and detailed comments, recommendations and questions. See our point-by-point response to the reviewer’s comments below.**

Thank you for allowing me to review this article. It is an interesting study using an interesting approach i.e. SP. I have a few comments:

 

- The conclusion in the abstract section must be consistent with the objective of the study and main findings.

 

**Thank you for this comment. The conclusion has been restructured to be consistent with the objectives of the study and main findings.**

 

- Percentages reported in tables must equal to 100% - kindly check all figures.

 

**Thank you for this comment. We have checked all percentages reported in tables and made corrections if necessary.**

 

- Tables cited in the main text should in proper order.

 

**Thank you for this comment. We have ensured that the tables are cited in the correct order.**

 

- Even though due to the nature of the study, subjects selected were not asked for consent, did the authors contact them after the study informing that a study was conducted with reasons? This is one way that the researchers could resolve the issue of informed consent prior to the study. 

 

**Thank you for this comment. Yes, we contacted the subjects after the study informing them that an SP study was conducted with reasons. Therefore, the following sentence and two additional references have been added: ‘To resolve the issue of informed consent, the authors contacted the pharmacies by mail and email after the study informing them that an SP study had been conducted with the corresponding background information ^48, 49^ .’**

 

- Create a paragraph on Study recommendations at the end of the Discussion section. Consider to move some parts of the conclusion. Rewrite the conclusion section.

 

**Thank you for this comment. We have created a new paragraph on study recommendations at the end of the discussion section and have moved some parts of the conclusion to this new paragraph. Additionally, the conclusions section has been rewritten.**
